# Studies of the Antiproliferative Activity of Ruthenium (II) Cyclopentadienyl-Derived Complexes with Nitrogen Coordinated Ligands

**DOI:** 10.1155/2010/936834

**Published:** 2010-06-20

**Authors:** Virtudes Moreno, Julia Lorenzo, Francesc X. Aviles, M. Helena Garcia, João P. Ribeiro, Tânia S. Morais, Pedro Florindo, M. Paula Robalo

**Affiliations:** ^1^Department de Química Inorgànica, Universitat de Barcelona, Martí y Franquès 1-11, 08028 Barcelona, Spain; ^2^Institut de Biotecnologia i de Biomedecina, Universitat Autònoma de Barcelona, Bellaterra, 08193 Barcelona, Spain; ^3^Centro de Ciências Moleculares e Materiais, Faculdade de Ciências da Universidade de Lisboa, Campo Grande, 1749-016 Lisboa, Portugal; ^4^Centro de Investigaciones Biológicas, CSIC, Ramiro de Maeztu 9, 28040 Madrid, Spain; ^5^Centro de Química Estrutural, Complexo I, Instituto Superior Técnico, Avenue Rovisco Pais, 1049-001 Lisboa, Portugal; ^6^Departamento de Engenharia Química, Instituto Superior de Engenharia de Lisboa, Avenue Conselheiro Emídio Navarro, 1, 1959-007 Lisboa, Portugal

## Abstract

Four cationic ruthenium(II) complexes with the formula [Ru(*η* ^5^-C_5_H_5_)(PPh_3_)_2_]^+^, with L = 5-phenyl-1H-tetrazole (TzH) **1**, imidazole (ImH) **2**, benzo[1,2-b;4,3-b′] dithio-phen-2-carbonitrile (Bzt) **3**, and [5-(2-thiophen-2-yl)-vinyl]-thiophene-2-carbonitrile] (Tvt) **4** were prepared and characterized in view to evaluate their potentialities as antitumor agents. Studies by Circular Dichroism indicated changes in the secondary structure of ct-DNA. Changes in the tertiary structure of pBR322 plasmid DNA were also observed in gel electrophoresis experiment and the images obtained by atomic force microscopy (AFM) suggest strong interaction with pBR322 plasmid DNA; the observed decreasing of the viscosity with time indicates that the complexes do not intercalate between DNA base pairs. Compounds **1**, **2**, and **3** showed much higher cytotoxicity than the cisplatin against human leukaemia cancer cells (HL-60 cells).

## 1. Introduction

The driving force for the studies of metal-based drugs in the treatment of cancer has been certainly the enormous impact of cisplatin. This platinum complex continues to be applied in 50%–70% of all cancer patients, usually in combination with other drugs [1]. Nevertheless, the undesirable side-effects of cisplatin, its inactivity against many cancer cell lines and tumors metastasis [2] has stimulated the search for new effective drugs, in the field of nonplatinum-based anticancer drugs [3–5]. Although the promising results for compounds derived of several metals such as titanium [6], iron [7], gold [8], gallium [9], and tin [10], it appears that the ruthenium derivatives occupy already a prominent position due to success of [ImH][trans-RuCl_4_(DMSO)Im], NAMI-A, [ImH][trans-RuCl_4_Im*_2_*] (Im = imidazole) and (Hind)[trans-RuCl_4_(ind)_2_], and KP1019, (ind = indazole) in progressing through clinical trials [11–13]. The main drawback of the coordination compounds concerning clinical trials has been related to their instability and complicated ligand exchange chemistry. Thus, organometallic field appeared as an attractive field to provide organoruthenium complexes as suitable drug candidates. Under this perspective, the studies already reported are quite promising, in some families of ruthenium *η* ^6^-arene derivatives which were found active against hypotoxic tumor cells [14, 15], in vitro breast and colon carcinoma cells [16, 17], inhibition of the growth of the human ovarian cancer cells line A2780 [18], and mammary cancer cell line [19]. Studies on *π*-arene compounds have been recently reviewed [20, 21]. Although the literature is scarce in studies concerning ruthenium *η* ^5^-cyclopentadienyl (RuCp) derivatives, it appears of interest the results already found for compounds containing the “CpRu(CO)” fragment and pyridocarbazole derived ligands which revealed to be potent and selective inhibitors for protein kinases GSK-3 and Pim-1 [22]. 

Our approach in this field has been the study of compounds derived of “RuCp” containing N-heteroaromatic sigma coordinated ligands [23] and CpRu(*η* ^6^-arene) where the structures of the *η* ^6^-arene ligands were based on two or three fused rings [24]. Our studies by AFM revealed a strong interaction with the plasmid pBR322 DNA. The obtained images showed compaction, supercoiling and kinks in the DNA forms. Circular Dichroism and viscosity measurements on ct-DNA also indicated that the complexes interact with the double helix. Compounds **1**, **2**, and **3** showed much higher cytotoxicity than the cisplatin against human leukaemia cancer cells (HL-60 cells). The compound **4** gave a lower IC_50_ value than cisplatin at 24 hours and a similar value at 72 hours. Moreover, some other CpRu compounds previously studied showed also significant effect of toxicity in Lovo and MiaPaCa cells in the nanomolar range [23].

## 2. Results and Discussion

Our continuing studies on the family of [Ru(*η* ^5^-C_5_H_5_)(PP)]^+^ fragment containing complexes in view of studies of cytotoxicity led us to the synthesis of two new cationic complexes of the type [Ru(*η* ^5^-C_5_H_5_)(PPh_3_)_2_ L]^+^ where the metal atom is *σ* bound to the donor nitrogen atom of the heteroaromatic ligands **L **= 5-phenyl-1H-tetrazole (**TzH**) and imidazole (**ImH**). 

Having in mind that different structures of the coordinated molecule **L**, can lead to different interactions with DNA, two other known compounds were also prepared and studied. One of these compounds presents the ligand **L** as a wide planar system based on a thiophene-benzene-thiophene ortho-fused rings structure, namely, benzo[1, 2-b; 4, 3-b′]dithio-phen-2-carbonitrile [25] (**Bzt**), while the ligand of the other compound is based on a long planar structure, namely, [5-(2-thiophen-2-yl)-vinyl]-thiophene-2-carbonitrile (**Tvt**) [26].

**Scheme 1:**
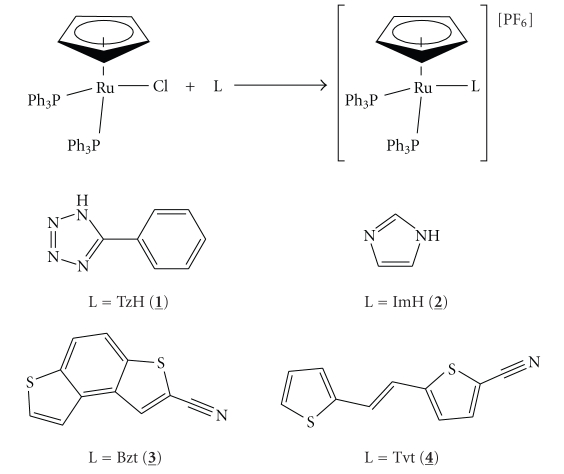
Reaction scheme for the synthesis of the [Ru(*η* ^5^-C_5_H_5_)(PPh_3_)_2_(L)][PF6] complexes **1**–**4 **and the structures of the ligands L.

The complexes [Ru(*η* ^5^-C_5_H_5_) (PPh_3_)_2_ L][PF_6_] with L = 5-phenyl-1H-tetrazole (**1**) and imidazole (**2**) were prepared by the general method of halide abstraction of [Ru(*η* ^5^-C_5_H_5_)(PPh_3_)_2_Cl] with thallium hexafluorophosphate in the presence of the adequate ligand (Scheme 1). The starting material [Ru(*η* ^5^-C_5_H_5_)(PPh_3_)_2_Cl] was prepared according to the literature [27] and the mentioned ligands were used as purchased. Compounds [RuCp(PPh_3_)_2_(Bzt)][PF_6_] **3** and [RuCp(PPh_3_)_2_(Tvt)][PF_6_] **4** and the corresponding ligands were prepared and characterised following our previous publications [25, 26]. 

Compounds were obtained in good yields and recrystallized by slow diffusion of diethyl ether in dichloromethane solutions. The new compounds were fully characterized by FT-IR, ^1^H, ^13^C, and ^31^P NMR spectroscopies; elemental analyses were in accordance with the proposed formulations.

### 2.1. Spectroscopic Studies

The main feature on the ^1^H NMR chemical shifts of the coordinated ligands is a general trend of shielding of the protons of the heteroaromatic rings with special relevance for the proton adjacent to the N coordinated atom. This effect, also observed on other related compounds [28], might be due to the influence of the organometallic moiety on the ring current of the heteroaromatic ligands. 

Interestingly, compound **2** shows one signal at 11.53 ppm, integrating perfectly for one proton and attributed to the NH proton of imidazole which, in the same solvent, could not be found in the free imidazole. This observation is in good agreement with the observed shielding on the other protons of the heteroaromatic ring upon coordination. Accordingly, ^13^C NMR spectra showed a general deshielding up to 13.6 ppm on the carbons of the coordinated ligand, this effect being more pronounced on compound **2**. 

The ^31^P NMR spectra revealed equivalency of both P atoms of the phosphines, with a singlet at ~42 ppm, with the expected deshielding upon coordination to the metal centre, and the septet relative to the counter ion PF_6_^−^ at −144 ppm.

The FT-IR spectra typically showed the characteristic bands of the Cp and Ph aromatic rings in the region 3040–3080 cm^−1^, and the characteristic bands of the PF_6_ anion at 840 and 560 cm^−1^. 

### 2.2. Electrochemical Studies

The electrochemical properties of the ligands and the new Ru(II) complexes **1** and **2** were examined by cyclic voltammetry in acetonitrile and dichloromethane solutions (1 × 10^−3^ M) using 0.2 M tetrabutylammonium hexafluorophosphate (TBAPF_6_) as supporting electrolyte. The redox potentials measured at the scan rate of 0.2 V/s are reported in Table 1. The electrochemical behaviour of complexes **3** and **4** was already reported [25, 26].

The cyclic voltammogram of TzH ligand is characterized by one irreversible redox process at *E*_pa_ = −0.69 V with the corresponding reductive wave at *E*_pc_ = −0.99 V in acetonitrile, being these values of −0.50 V and −0.80 V, respectively, in dichloromethane. Due to the insolubility of ImH in dichloromethane, its electrochemistry was only studied in acetonitrile and revealed two irreversible processes, one oxidation at *E*_pa_ = +1.42 V without the cathodic counterpart and one reduction at *E*_pc_ = −0.88 V without the corresponding anodic complement. 

**Figure 1:**
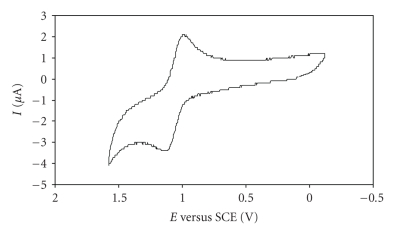
Cyclic voltammogram of compound **2** in dichloromethane at sweep rate of 200 mV/s.

 Both complexes were electroactive in the sweep range ±1.8 V and displayed one-electron quasi-reversible coupled redox wave in dichloromethane solution, as showed in Figure 1. The oxidative couple exhibited by the complexes **1 **and **2** with *E *_1/2_ at 1.24 and 1.06 V, respectively, is attributed to the Ru(II)/Ru(III) redox process in accordance with our earlier results in some analogous ruthenium compounds [25, 26, 29], these values being lower than those found for complexes **3** and **4**. At negative potentials, it was not found any redox process that could be related with the ligands. During the electrochemical study of complex **1** some passivation occurred at the platinum electrode, probably due to any potential-induced polymerisation processes in this solvent, originated by the ligand. 

The electrochemical behaviour of the studied complexes in acetonitrile was quite different. The cyclic voltammograms were characterised by one first irreversible oxidation process at *E*_pa_ = 0.86 V and *E*_pa_ = 0.92 V for complexes **1** and **2** appearing a second irreversible oxidation process at *E*_pa_ = 1.22 V for complex **1** and *E*_pa_ = 1.25 V for complex **2**.

By analogy with the electrochemistry run in dichloromethane, this second anodic wave was attributed to the oxidation Ru^II^/Ru^III^ for the present studied complexes. In order to identify the species responsible for the first oxidation process, some additional experiments were carried out. It was postulated that the most likely candidate would be the cationic complex [RuCp(PPh_3_)_2_(NCMe)]^+^, originated in the electrochemical cell, by substitution of the 5-phenyl-1H-tetrazole and imidazole ligands by the acetonitrile coordinative solvent.

Experiments in the nmr tube, with a sample solution of compound **2** in CD_3_CN revealed a slow substitution of the imidazole ligand by the solvent. In effect, only few hours later a significant amount of the new compound was found in the ^1^H and ^31^P NMR nmr spectra, being a conversion of about 90% found only 72 hours later. Although this slow substitution process is not likely to occur at the electrochemistry time scale, the electrochemistry of compound [RuCp(PPh_3_)_2_(NCMe)][PF_6_], prepared in the bench for this purpose, was studied in the same experimental conditions of compounds **1** and **2** to discard this hypothesis. The voltammogram only revealed one oxidative process occurring at ~*E*_pa_ = 1.48 V, attributed to the oxidation Ru^II^/Ru^III^. 

Although no further experiments were carried out, it seems that one possible explanation for the waves occurring at *E*_pa_ = 0.86 V and *E*_pa_ = 0.92 V for complexes **1** and **2**, can be an irreversible oxidation occurring at the coordinated ligands. 

The ligand-based reduction waves were found at *E*_pc_ = −0.70 V and −0.47 V for complexes **1** and **2**, respectively, while for the corresponding free ligands the values were *E*_pc_ = −0.99 V and −0.88 V. Thus, the corresponding potentials were in the order TzH < ImH < **1** < **2**, with the ruthenium complexes showing the most facile reductions. The stability of the pair Ru^II^/Ru^III^ showed by the electrochemical studies carried out in dichloromethane suggests the possible existence of Ru(III) analogues of compounds **1** and **2**which interest might betheir potentiality as bio reductive prodrugs.

## 3. Biological Activity

### 3.1. Circular Dichroism

**Figure 2:**
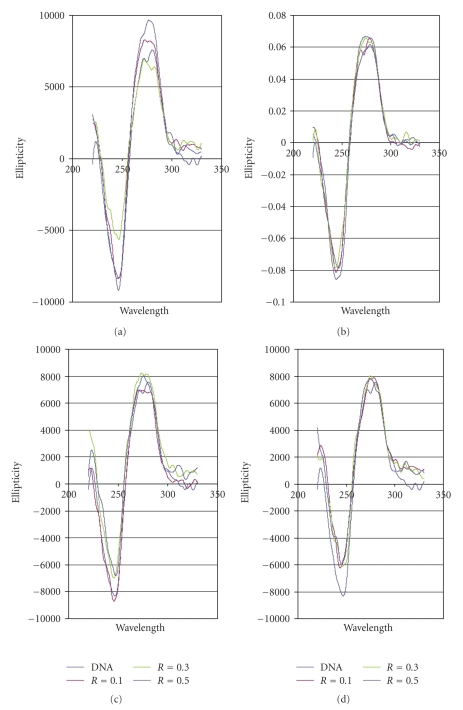
Circular Dichroism spectra of ct-DNA incubated with the complexes at molar ratios 0.1, 0.3, and 0.5, at 37°C for 24 hours (a) complex **1**, (b) complex **2**, (c) complex **3**, and (d) complex **4**.

In Figures 2(a)–2(d), the circular dichroism spectra of the compounds at several molar ratio ruthenium complex DNA are showed. After 24 hours of incubation at 37°C, changes in molar ellipticity can be observed for the complexes **1**, **2**, **3**, and **4**. These changes in the wavelength and the ellipticity of free DNA indicate modifications on the secondary structure of DNA as consequence of the interaction of the complexes with DNA. In spite of the substitution of ligands by N atoms of DNA bases could be possible, the most probable hypothesis is to consider weak interactions of the ligands with DNA through hydrogen bonds. 

### 3.2. Atomic Force Microscopy (AFM)

**Figure 3:**
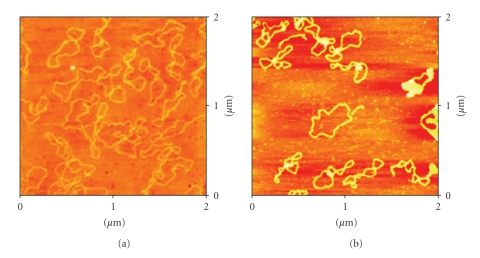
AFM image of the (a) free plasmid pBR322 DNA, and (b) plasmid pBR322 DNA incubated with the complex [RuCp(PPh_3_)_2_(TzH)][PF_6_] **1** *r*_*i*_ = 0.5.

**Figure 4:**
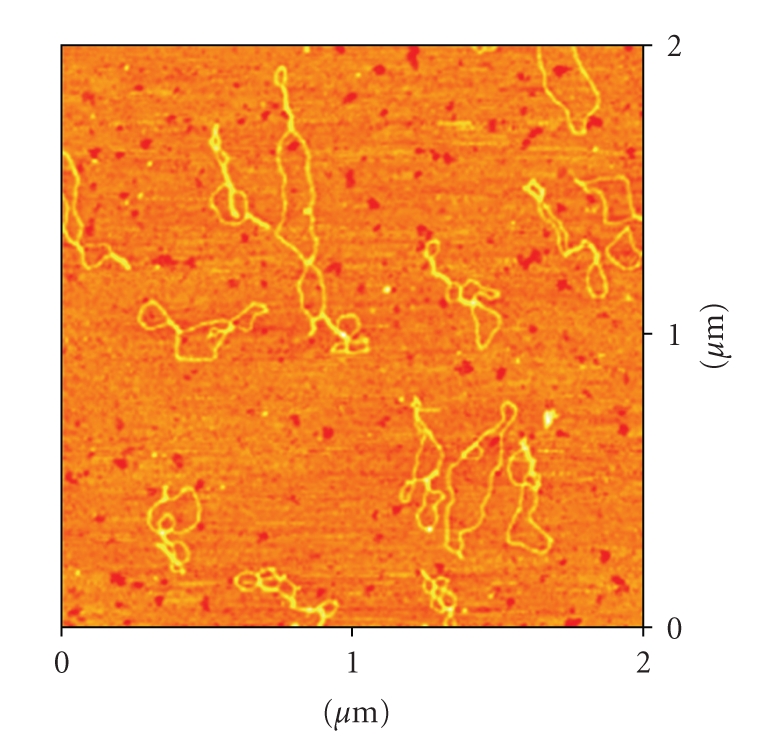
AFM image of the plasmid pBR322 DNA incubated with the complex [RuCp(PPh_3_)_2_(ImH)][PF_6_] **2** *r*_*i*_ = 0.5.

**Figure 5:**
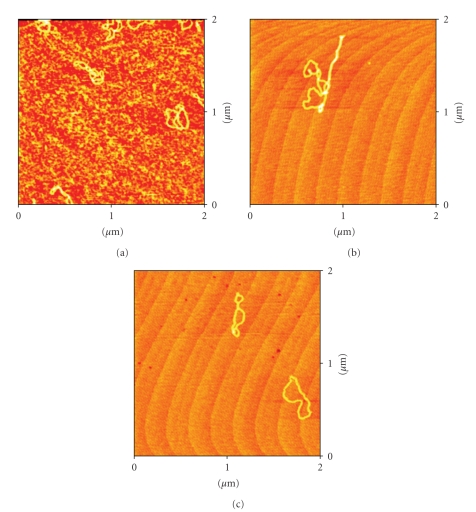
Three AFM images (different 2 × 2 *μ*m areas on mica sufarce) of the plasmid pBR322 DNA incubated with the complex [RuCp(PPh_3_)_2_(Bzt)][PF_6_] **3** *r*_*i*_ = 0.5.

**Figure 6:**
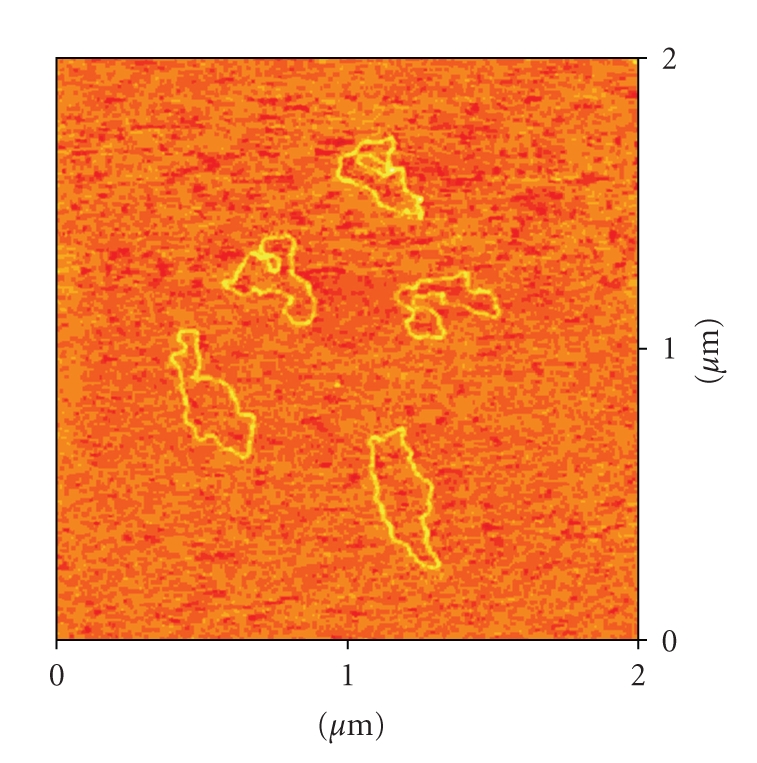
AFM image of the plasmid pBR322 DNA incubated with the complex [RuCp(PPh_3_)_2_(Tvt)][PF_6_] **4** *r*_*i*_ = 0.5.

AFM pictures of DNA pBR322, and DNA incubated with the complexes **1**–**4** are shown in Figures 3–6.

The AFM image (Figure 3) corresponding to DNA incubated with compound **1** in comparison with free DNA shows typical modifications in the DNA forms, supercoiling, kinks and compaction. Compound **2** (Figure 4) also modifies DNA forms, although the appearance of kinks is more evident. In the case of compounds with the ligands Bzt and Tvt, compounds **3** and **4** (Figures 5 and 6, resp.) the interaction with DNA is stronger. In the case of compound **3**, the first image shows some DNA forms affected by supercoiling and kinks. In the two other images only a few forms can be observed but in them strong modification can be appreciated. Finally, in the case of compound **4**, the image shows several DNA forms with kinks and microfolds. These modifications in comparison to the free pBR322 indicate that the four compounds interact with DNA. Additional measurements of the variation of viscosity with time at constant temperature show a decreasing of the viscosity, what allow us to conclude that there is not intercalation of the ligands between base pairs of DNA. (See supplementary material available online at doi: 10.1155/2010/963834.)

### 3.3. Electrophoretic Mobility

The influence of the compounds on the tertiary structure of DNA was determined by its ability to modify the electrophoretic mobility of the covalently closed circular (*ccc*) and open (*oc*) forms of pBR322 plasmid DNA. Figure 4 shows, from right to left, the mobility of the native pBR322 plasmid DNA and the plasmid DNA incubated with cisplatin as a reference, and with the compounds **1**–**4**. The behavior of the gel electrophoretic mobility of pBR322 plasmid and DNA ruthenium compounds adducts show changes compatible with the AFM images. In the pattern corresponding to the pBR322 plasmid DNA (lane 6) a clean difference between the OC and CCC bands can be observed. In lane 5, the typical coalescence of the two bands for cisplatin is evident. In the zone between OC and CCC bands in lanes 1–4 corresponding to the DNA incubated with ruthenium complexes, new bands (lane 4, compound **4**) or one continuous band (lanes 1–3, compounds **1**–**3**) appear indicating the presence of DNA forms with different mobility in agarose gel.

### 3.4. Cytotoxicity of the Ruthenium Complexes against HL-60 Cells

The effect of the ruthenium complexes was examined on human leukaemia cancer cells (HL-60) using the MTT assay, a colorimetric determination of cell viability during in vitro treatment with a drug. The assay, developed as an initial stage of drug screening, measures the amount of MTT reduction by mitochondrial dehydrogenase and assumes that cell viability (corresponding to the reductive activity) is proportional to the production of purple formazan that is measured spectrophotometrically. A low IC_50_ is desired and implies cytotoxicity or antiproliferation at low drug concentrations.

The drugs tested in this experiment were cisplatin, [Ru(*η* ^5^-C_5_H_5_)(PPh_3_)_2_TzH]^+^ **1**, [Ru(*η* ^5^-C_5_H_5_)(PPh_3_)_2_ ImH]^+^ **2**, [Ru(*η* ^5^-C_5_H_5_)(PPh_3_)_2_ Bdt]^+^ **3**, and [Ru(*η* ^5^-C_5_H_5_)(PPh_3_)_2_ Tvt]^+^ **4**.

**Figure 8:**
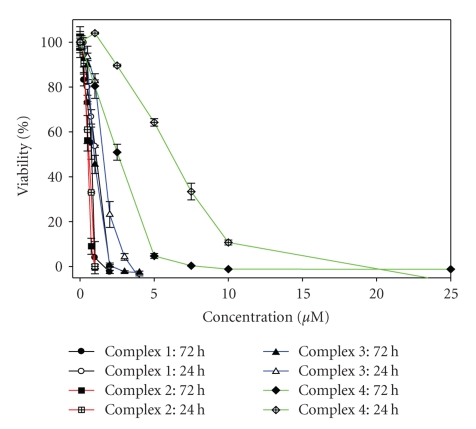
Inhibitory effects of complexes **1**, **2**, **3** and **4** on tumor cell proliferation in vitro.

Cells were exposed to each compound continuously for a 24 hours or a 72 hours period of time and then assayed for growth using the MTT endpoint assay. Figure 8 shows the dose-response curves of these drugs in terms of the drug effect on the growth of the HL-60 cells. The IC_50_ values of complex **1**, complex **2**, complex **3**, complex **4** and cisplatin for the growth inhibition of HL-60 cells were summarized in Table 2.

All of these ruthenium complexes exhibited antitumor effect against HL-60 cells. It was notable that complex **4** exerted the least potent effect among all the complexes, but this effect is better than that of cisplatin at 24 hours and the IC_50_ are very similar at 72 hours. The cytotoxicities displayed by **1**, **2**, and **3** were comparable with **3** slightly over **1** and **2**. 

### 3.5. Quantification of Apoptosis by Annexin V Binding and Flow Cytometry

We have also analysed by Annexin V-PI flow cytometry whether complexes **1**, **2**, **3**, and **4** are able to induce apoptosis in HL-60 cells after 24 hours of incubation at equitoxic concentrations (IC_50 _ values). Annexin V binds phosphatidyl serine residues, which are asymmetrically distributed towards the inner plasma membrane but migrate to the outer plasma membrane during apoptosis [30].

As it can be seen in Table 3, all metal complexes induce cell death mainly by apoptosis. Complexes **1** and **2** are less toxic than complexes **3** and **4**, the first two complexes induced less necrotic or damage cells at IC_50_ treatment.

## 4. Conclusion

The complexes were tested for potential antitumor activity against the human promyelocytic leukemia cell line HL-60 using a MTT assay. The four complexes tested possess excellent antitumor activities, with IC_50_ values lower than that of cisplatin. It cannot be discarded that one of the targets of the antitumor process would be the DNA, because interaction of the four compounds with DNA(ct-DNA or pBR322 plasmid DNA) has been demonstrated by CD, Electrophoretic mobility, and AFM studies. 

## 5. Experimental Protocols

All the syntheses were carried out under dinitrogen atmosphere using current Schlenk techniques and the solvents used were dried using standard methods [31]. The starting material [Ru(*η* ^5^-C_5_H_5_)(PPh_3_)_2_Cl] was prepared following the method described in the literature [27]. Compounds [RuCp(PPh_3_)_2_(Bzt)][PF_6_] **3** and [RuCp(PPh_3_)_2_(Tvt)][PF_6_] **4** were previously characterised [25, 26]. FT-IR spectra were recorded in a Mattson Satellite FT-IR spectrophotometer with KBr pellets; only significant bands are cited in text. ^1^H-, ^13^C-, and ^31^P-NMR spectra were recorded on a Bruker Advance 400 spectrometer at probe temperature. The ^1^H and ^13^C chemical shifts are reported in parts per million (ppm) downfield from internal Me_4_Si and the ^31^P NMR spectra are reported in ppm downfield from external standard, 85% H_3_PO_4_. Elemental analyses were obtained at Laboratório de Análises, Instituto Superior Técnico, using a Fisons Instruments EA1108 system. Data acquisition, integration and handling were performed using a PC with the software package EAGER-200 (Carlo Erba Instruments). Electronic spectra were recorded at room temperature on a *Jasco V-560* spectrometer in the range of 200–900 nm. 

### 5.1. Synthesis of [CpRu(PPh_3_)_2_(TzH)][PF_6_] (1)


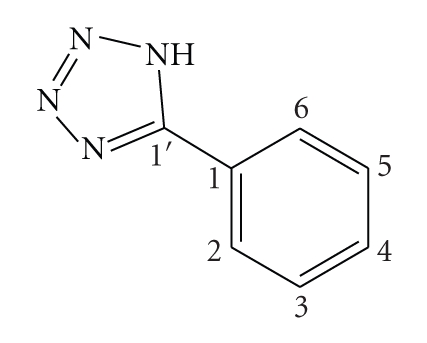


To a suspension of [CpRu(PPh_3_)_2_Cl] (0.73 g; 1 mmol) in THF : CH_2_Cl_2_ (3 : 1) was added 5-phenyl-1H-tetrazole (0.15 g; 1.1 mmol) followed by the addition of TlPF_6_ (0.35 g; 1 mmol). The orange mixture was slightly warmed at 35°C during 30 minutes and turned yellow. The precipitate of TlCl was removed by cannula-filtration and the solvent evaporated. The yellow oil residue was treated with n-hexane and the obtained solid was recrystallized from dichloromethane/ethyl ether. Yield: 80 %.

^1^H NMR [CDCl_3_, Me_4_Si, *δ*/ppm]: 7.70 [*d*, 2, H_2_ + H_6_(TzH); J_2,6_= 7.7 Hz]; 7.50–7.44 [*m,* 3, H_3_ + H_4_+ H_5_(TzH)]; 7.35 [*t*, 6, H_para_ (PPh_3_)]; 7.24 [*t*, 12, H_meta_ (PPh_3_)]; 7.17 [*d*, 12, H_orto_ (PPh_3_) ]; 4.46 [*s*, 5, *η* ^*η*5^-C_5_H_5_].

^13^C NMR [CDCl_3_, *δ*/ppm]: 156.95 (C_1_, TzH); 136.82 (Cq, PPh_3_); 134.07 (C_4_, TzH); 132.16 (CH, PPh_3_); 130.26 (C_3_ + C_5_, TzH); 129.70 (CH, PPh_3_); 125.45 (CH, PPh_3_); 128.07 (C_2_ + C_6_, TzH); 127.72 (C_1_, TzH); 83.47 (C_5_H_5_). 

^31^P NMR [CDCl_3_, *δ*/ppm]: 41.9 [*s*, PPh_3_]; −144.1 [*sept*, PF_6_^−^].

FT-IR [KBr, cm^−1^]: 3057 (wm); 2984 (w); 2913 (w); 2836 (w); 2760 (w); 2699 (w); 2609 (wm); 2550 (w); 2481 (w); 1608 (w); 1563 (wm); 1481 (m); 1434 (ms); 1410 (m); 1312 (w); 1185 (w); 1163 (w); 1089 (ms); 997 (m); 840 (s); 745 (ms); 697 (s); 557 (ms); 521 (S); 496 (ms); UV-Vis. in CH_2_Cl_2_, *λ*_max _ /nm (*ε*/M^−1^cm^−1^): 250 (21176), 348 (7603); Elem. Anal. Found: C 58.93; H 4.21; N 5.70; Calc. for C_48_H_41_N_4_P_3_F_6_Ru; C, 58.72; H, 4.21; N, 5.71. 

### 5.2. Synthesis of [CpRu(PPh_3_)_2_(ImH)][PF_6_] (2)


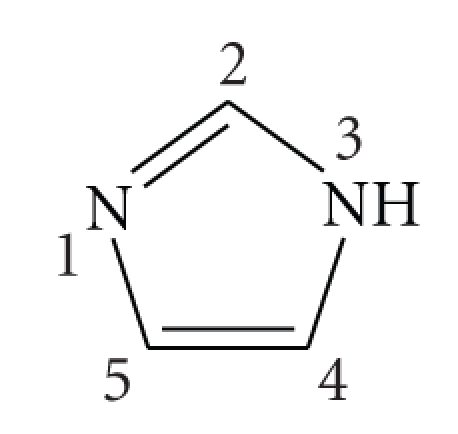


To a solution of [CpRu(PPh_3_)_2_Cl] (0.20 g; 0.275 mmol) in dichloromethane (25 mL), imidazole was added (0.02 g; 0.3 mmol) followed by the addition of TlPF_6_ (0.11 g; 0.3 mmol). The reaction was carried out at room temperature with vigorous stirring during 14 hours with the change of colour from orange to yellow. The precipitate of TlCl was removed by cannula-filtration and the solvent evaporated. The product was washed with n-hexane and recrystallized from dichloromethane/ethyl ether. Yield: 70%.

^1^H NMR [(CD_3_)_2_CO, Me_4_Si, *δ*/ppm]: 11.53 [s, 1, NH(Im)]; 7.46 [*t*, 6, H_para_ (PPh_3_)]; 7.37 [*t*, 12, H_meta_ (PPh_3_)]; 7.23 [*d*, 12, H_ortho_ (PPh_3_)]; 4.57 [*s*, 5, *η* ^5^-C_5_H_5_], H_2_ + H_4_ + H_5_ (Im) obscured by PPh_3_.^ 13^C NMR [(CD_3_)_2_CO, *δ*/ppm]: 144.2 (C_2_, Im); 135.5 (C_4_ +C_5_, Im); 133.61 (PPh_3_); 133.55 (PPh_3_); 133.50 (PPh_3_); 130.0 (PPh_3_); 128.42 (PPh_3_); 128.38 (PPh_3_); 128.33; 117.8 (Im); 82.6 (Cp); ^31^P NMR [(CD_3_)_2_CO, *δ*/ppm]: 42.04 (*s*, PPh_3_); −144.04 (*sept*, PF_6_^−^).

FT-IR [KBr, cm^−1^]: 3056 (w); 2955 (w); 1480 (m); 1434 (s); 1089 (m); 999 (w); 840 (s); 745 (s); 696 (S); 557 (s); 522 (s).

UV-Vis. in CH_2_Cl_2_, *λ*_max _/nm (*ε*/M^−1^cm^−1^): 248 (21200), 273 (8162), 348 (3285); Elem. Anal. Found: C, 58.19; H, 4.68; N, 3.16. Found for C_44_H_39_N_2_P_3_F_6_Ru: C, 58.47; H, 4.35; N, 3.10.

### 5.3. Electrochemical Experiments

Cyclic voltammograms of ligands and complexes were obtained using a EG and G Princeton Applied Research Model 273A potentiostat/galvanostat monitored with a personal computer loaded with Electrochemistry PowerSuite v2.51 software from Princeton Applied Research at room temperature. A three-electrode configuration small capacity cell was equipped with a platinum-disk working electrode (1.0 mm diameter), a silver-wire pseudo-reference electrode connected by a Lugging capillary and a platinum wire auxiliary electrode. The electrochemical experiments were performed in 0.2 M solutions of TBAPF_6_ in dichloromethane or acetonitrile, under a nitrogen atmosphere and the redox potentials were measured using ferrocene as the internal standard. The redox potential values were quoted relative to the SCE by using the ferrocenium/ferrocene redox couple (*E*_*p*/2_ = 0.46 or 0.40 V versus SCE for dichloromethane or acetonitrile, resp.) [32].

The supporting electrolyte was purchased from Aldrich Chemical Co., recrystallized from ethanol, washed with diethyl ether and dried under vacuum at 110°C for 24 hours. Reagent grade acetonitrile and dichloromethane were dried over P_4_O_10_ and CaH_2_, respectively, and distilled under nitrogen atmosphere before use.

### 5.4. Biological Assays

#### 5.4.1. DNA Interaction Studies

**Circular Dichroism**

All compounds were dissolved in an aqueous solution (prepared with milli-Q water) of 4% DMSO (2 mg compound/5 mL). The stock solutions were freshly prepared before use. The samples were prepared by addition of aliquots of these stock solutions to the appropriate volume of Calf Thymus DNA in a TE buffer solution (50 mM NaCl, 10 mM tris-(hydroxymethyl)aminomethane hydrochloride (Tris-HCl), 0.1 mM H_4_edta, pH 7.4) (5 mL). The amount of complex added to the DNA solution was designated as *r*_*i*_ (the input molar ratio of Ru to nucleotide and it is calculated with formula

(1) $$r_{i}=\frac{m\times M_{\text{nucl}}\times Am}{C\times Mr\times V},$$

where *m* = mass of the compound (*μ*g); *M*_nucl_ = medium nuclear mass per nucleotide (330 g/mol); *C* = concentration of the DNA solution (*μ*g/ml); *Mr* = molecular mass of each compound (g/mol); *V* = total volume of each sample (5 mL).As a blank, a solution in TE of free native DNA was used. The CD spectra of DNA in the presence or absence of complexes (DNA concentration 20 *μ*g/mL, molar ratios *r_i_* = 0.10, 0.30, 0.50) were recorded at room temperature, after 24 hours incubation at 37°C, on a JASCO J-720 spectropolarimeter with a 450 W xenon lamp using a computer for spectral subtraction and noise reduction. Each sample was scanned twice in a range of wavelengths between 220 and 330 nm. The CD spectra drawn are the average of three independent scans. The data are expressed as average residue molecular ellipticity (*θ*) in degrees·cm^2^·dmol^−1^.

**Viscosity Measurements**

Viscosity experiments were carried out with an AND-SV-1 viscometer in a water bath using a water jacket accessory and maintained the constant temperature at 25°C. A range of 200–370 *μ*L of 5 mM solutions of the different compounds were added in 2 mL of 100 mM ct-DNA solution. The flow time was measured by a digital stop watch.

**Atomic Force Microscopy (TMAFM)**

DNA pBR322 was heated at 60° for 10 minutes to obtain OC form. Stock solution was 1 mg/mL in a buffer solution of HEPES (4 mM Hepes, pH 7.4/2 mM MgCl_2_). Each sample contained 1 *μ*L of DNA pBR322 of concentration 0.25 *μ*g/ *μ*L for a final volume of 40 *μ*L. The amount of drug added is also expressed as *r_i_*. AFM samples were prepared by casting a 3-*μ*L drop of test solution onto freshly cleaved Muscovite green mica disks as the support. The drop was allowed to stand undisturbed for 3 minutes to favour the adsorbate-substrate interaction. Each DNA-laden disk was rinsed with Milli-Q water and was blown dry with clean compressed argon gas directed normal to the disk surface. Samples were stored over silica prior to AFM imaging. All Atomic Force Microscopy (AFM) observations were made with a Nanoscope III Multimode AFM (Digital Instrumentals, Santa Barbara, CA). Nano-crystalline Si cantilevers of 125-nm length with a spring constant of 50 N/m average ended with conical-shaped Si probe tips of 10-nm apical radius and cone angle of 35° were utilized. High-resolution topographic AFM images were performed in air at room temperature (relative humidity < 40%) on different specimen areas of 2 × 2 *μ*m operating in intermittent contact mode at a rate of 1–3 Hz.

**Gel Electrophoresis of Ruthenium Complexes-pBR322**

pBR322 DNA aliquots (0.25 *μ*g/mL) were incubated in TE buffer (10 mM Tris.HCl, 1 mM EDTA, pH = 7,5) at molar ratio *r_i_* = 0.50 for electrophoresis study. Incubation was carried out in the dark at 37°C for 24 hours. 4 *μ*L of charge marker were added to aliquots parts of 20 *μ*L of the compound-DNA complex. The mixture was electrophoresedin agarose gel (1% in TBE buffer, Tris-Borate-EDTA) for 5 hours at 1.5 V/cm. Afterwards, the DNA was dyed with ethydium bromide solution (0.75 *μ*g/mL in TBE) for 6 hours. A sample of free DNA was used as control. The experiment was carried out in an ECOGEN horizontal tank connected to a PHARMACIA GPS 200/400 variable potential power supply and the gel was photographed with an image Master VDS, Pharmacia Biotech.

### 5.5. Tumor Cell Lines and Culture Conditions

The cell line used in this experiment was the human acute promyelocytic leukaemia cell line HL-60 (American Type Culture Collection (ATCC)). Cells were routinely maintained in RPMI-1640 medium supplemented with 10% (v/v) heat inactivated foetal bovine serum, 2 mmol/L glutamine, 100 U/mL penicillin, and 100 *μ*g/mL streptomycin (Gibco BRL, Invitrogen Corporation, Netherlands) in a highly humidified atmosphere of 95% air with 5% CO_2_ at 37°C.

**Cytotoxicity Assays**

Growth inhibitory effect of ruthenium complexes on the leukaemia HL-60 cell line was measured by the microculture tetrazolium, [3-(4,5-dimethylthiazol-2-yl)-2,5-diphenyltetrazolium bromide, MTT] assay [33]. Briefly, cells growing in the logarithmic phase were seeded in 96-well plates (10^4^ cells per well), and then were treated with varying doses of ruthenium complexes and the reference drug cisplatin at 37°C for 24 or 72 hours. For each of the variants tested, four wells were used. Aliquots of 20 *μ*L of MTT solution were then added to each well. After 3 hours, the colour formed was quantified by a spectrophotometric plate reader at 490 nm wavelength. The percentage cell viability was calculated by dividing the average absorbance of the cells treated with a platinum complex by that of the control; IC_50_ values (drug concentration at which 50% of the cells are viable relative to the control) were obtained by GraphPad Prism software, version 4.0.

**In Vitro Apoptosis Assay**

Induction of apoptosis in vitro by ruthenium compounds was determined by a flow cytometric assay with Annexin V-FITC by using an Annexin V-FITC Apoptosis Detection Kit (Roche) [30]. Exponentially growing HL-60 cells in 6-well plates (5 × 10^5^ cells/well) were exposed to concentrations equal to the IC_50_ of the ruthenium drugs for 24 hours. Afterwards, the cells were subjected to staining with the Annexin V-FITC and propidium iodide. The amount of apoptotic cells was analyzed by flow cytometry (BD FACSCalibur).

## Supplementary Material

Variation of viscosity with time at constant temperature for complexes [RuCp(PPh_3_)_2_(Bzt)][PF_6_] **3** and [RuCp(PPh_3_)_2_(Tvt)][PF_6_] **4**

## Figures and Tables

**Figure 7:**
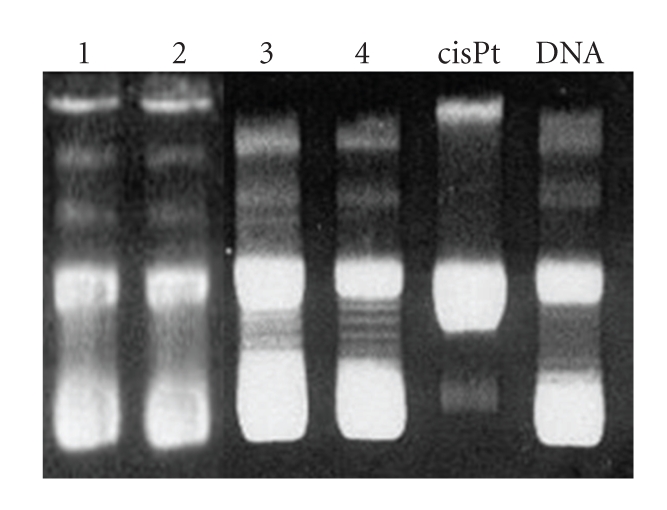
Agarose gel electrophoretic mobility of DNA pBR322 treated with compounds **1** (lane 1); compound **2** (lane 2); compound **3** (lane 3); compound **4** (lane 4); **cisplatin** (lane 5) and DNA pBR322 (lane 6).

**Table 1:** Electrochemical data for the [RuCp(PPh_3_)_2_L)][PF_6_] (L = TzH, ImH) complexes and the free ligands in dichloromethane and acetonitrile.

Dichloromethane					
Compound	*E*_pa_ (V)	*E*_pc_ (V)	*E *_1/2_ (V)	Δ*E^a^* (mV)	*I*_*c*_/*I*_*a*_

**TzH**	−0.50	−0.80	—	—	—
**ImH^b^**	—	—	—	—	—
**1 **	1.27	1.21	1.24	60	1.0
**2 **	1.10	1.02	1.06	80	1.0

Acetonitrile					
Compound	*E*_pa_ (V)	*E*_pc_ (V)	*E *_1/2_ (V)	Δ*E^a^* (mV)	*I*_*c*_/*I*_*a*_

**TzH**	−0.69	−0.99	—	—	—
**ImH**	1.42	—	—	—	—
	—	−0.88	—	—	—
**1**	1.22	—	—	—	—
	0.86	—	—	—	—
	—	−0.70	—	—	—
**2 **	1.25	—	—	—	—
	0.92	—	—	—	—
	—	−0.47	—	—	—

^a^Δ*E* = *E*_pa_ − *E*_pc_; ^b^Insoluble in dichloromethane.

**Table 2:** IC_50_ values of ruthenium compounds, and cisplatin against HL-60 cells.

Complex	IC_50_ (*μ*M) 72 hours	IC_50_ (*μ*M) 24 hours
[Ru(*η* ^5^-C5H_5_)(PPh_3_)_2_TzH]^+^ **1**	0.69 ± 0.16	0.95 ± 0.15
[Ru(*η* ^5^-C5H_5_)(PPh_3_)_2_ImH]^+^ **2**	0.53 ± 0.05	0.57 ± 0.09
[Ru(*η* ^5^-C5H_5_)(PPh_3_)_2_Bdt]^+^ **3**	0.94 ± 0.085	1.46 ± 0.25
[Ru(*η* ^5^-C5H_5_)(PPh_3_)_2_Tvt]^+^ **4**	2.26 ± 0.53	5.89 ± 0.67
CDDP	2.15 ± 0.1	15.61 ± 1.15

**Table 3:** Percentage of HL-60 cells in each state after treatment with metal complexes at IC_50_ concentration for 24 hours of incubation.

Treatment (IC_50_ 24 hours *μ*M)	% vital cells (R1)	% apoptotic cells (R2)	% dead cells (R3)	% damaged cells (R4)
Control	91.1	4.8	3.9	0.2
CDDP	55.0	40.7	3.6	0.7
[Ru(*η* ^5^-C5H_5_)(PPh_3_)_2_TzH]^+^ **1**	74.7	19.9	5.0	0.4
[Ru(*η* ^5^-C5H_5_)(PPh_3_)_2_ImH]^+^ **2**	62.6	29.8	7.3	0.3
[Ru(*η* ^5^-C5H_5_)(PPh_3_)_2_Bdt]^+^ **3**	60.0	20.8	13.3	5.9
[Ru(*η* ^5^-C5H_5_)(PPh_3_)_2_Tvt]^+^ **4**	51.4	18.1	21.0	9.5
