# Supplementary figures and images for: Studies of the Antiproliferative Activity of Ruthenium (II) Cyclopentadienyl-Derived Complexes with Nitrogen Coordinated Ligands
Source: Bioinorg Chem Appl. 2010 Jun 20;2010:936834. doi: 10.1155/2010/936834 (PMC2905958; doi:10.1155/2010/936834)

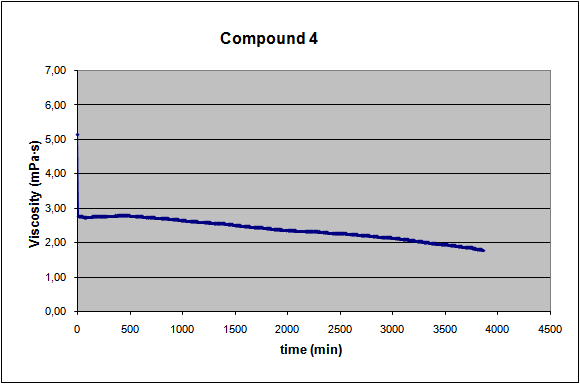

Supplement: Supplementary file 1 — Variation of viscosity with time at constant temperature for complexes [RuCp(PPh3)2(Bzt)][PF6] 3 and [RuCp(PPh3)2(Tvt)][PF6] 4 [file 936834.f1.tif]
